# Metabolites of prostaglandin synthases as potential biomarkers of Lyme disease severity and symptom resolution

**DOI:** 10.1007/s00011-018-1180-5

**Published:** 2018-08-18

**Authors:** Alicia Caroline Jarosz, Alaa Badawi

**Affiliations:** 1 Department of Nutritional Sciences, Faculty of Medicine, University of Toronto, FitzGerald Building, 150 College Street, Toronto, ON M5S3E2 Canada; 2 Public Health Risk Sciences Division, Public Health Agency of Canada, 180 Queen Street West, Toronto, ON M5V 3L7 Canada

**Keywords:** Systematic review, Lyme disease, *Borrelia burgdorferi*, Prostaglandins, COX, Arachidonic acid, Leukotrienes, Thromboxanes, Non-steroidal anti-inflammatory drugs

## Abstract

**Background:**

Lyme disease or Lyme borreliosis (LB) is the commonest vector-borne disease in the North America. It is an inflammatory disease caused by the bacterium *Borrelia burgdorferi*. The role of the inflammatory processes mediated by prostaglandins (PGs), thromboxanes and leukotrienes (LTs) in LB severity and symptoms resolution is yet to be elucidated.

**Objectives:**

We aim to systematically review and evaluate the role of PGs and related lipid mediators in the induction and resolution of inflammation in LB.

**Methods:**

We conducted a comprehensive search in PubMed, Ovid MEDLINE(R), Embase and Embase Classic to identify cell-culture, animal and human studies reporting the changes in PGs and related lipid mediators of inflammation during the course of LB.

**Results:**

We identified 18 studies to be included into this systematic review. The selected reports consisted of seven cell-culture studies, seven animal studies, and four human studies (from three patient populations). Results from cell-culture and animal studies suggest that PGs and other lipid mediators of inflammation are elevated in LB and may contribute to disease development. The limited number of human studies showed that subjects with Lyme meningitis, Lyme arthritis (LA) and antibiotic-refractory LA had increased levels of an array of PGs and lipid mediators (e.g., LTB_4,_ 8-isoPGF_2α_, and phospholipases A_2_ activity). Levels of these markers were significantly reduced following the treatment with antibiotics or non-steroidal anti-inflammatory drugs.

**Conclusion:**

Dysregulation of prostaglandins and related lipid mediators may play a role in the etiology of LB and persistence of inflammation that may lead to long-term complications. Further investigation into the precise levels of a wide range of PGs and related factors is critical as it may propose novel markers that can be used for early diagnosis.

**Electronic supplementary material:**

The online version of this article (10.1007/s00011-018-1180-5) contains supplementary material, which is available to authorized users.

## Introduction

Lyme disease—also known as Lyme borreliosis (LB)—can be caused in humans by at least three genospecies of the *Borrelia burgdorferi* sensu lato complex; *B. burgdorferi, B. garinii* and *B. afzelii*. In the northern part of the United States and southern Canada, *B. burgdorferi* sensu stricto cause flu-like illness at early disease stages that can later develop to Lyme arthritis and other long-term complications. In Eurasia, however, *B. garinii* and *B. afzelii* are predominant and can lead to neurological and skin complications [1]. LB is an inflammatory disease initiated by the bacterial infection following a bite from an infected *Ixodes scapularis*, and *Ixodes pacificus* blacklegged ticks. At present, LB is the most common vector-borne disease in the North America and Europe [1]. Over 30,000 cases in the US are reported annually [2]. In Canada, an increased incidence of LB by ~ sixfold—from 128 to 707 cases—was noted between 2009 and 2015 [3]. However, actual prevalence estimates are thought to be at least ten times as high because of the underreporting, as the cases are usually only captured if acquired in known endemic areas, and the dependence on insensitive diagnostic tests, particularly at the early disease stage [4].

Symptoms of early stages of LB include erythema migrans (EM) with or without inflammatory reactions including fever, chills and malaise. Disseminated infection may occur early or late along the natural history of disease development and can involve the skin, musculoskeletal and nervous system [5]. As the innate and adaptive immune responses develop following the infection, patients may resolve the early disease symptoms with or without antibiotic therapy. However, a significant proportion of LB patients treated with antibiotics do not exhibit detectable antibodies on convalescent testing [6, 7] and are subject to develop persistent or post-treatment Lyme disease symptoms (PTLDS). Among other complications, PTLDS include acute or persistant arthritis, meningitis, neuroborreliosis and myocarditis, a rare sequela that may lead to death [8–11]. The proportion of LB patients with PTLDS varies greatly, from 0 to 50%, depending on the population and the case definition [7].

The clinical manifestations of LB are attributed primarily to the host’s immune response to infection [12]. Host infection with *B. burgdorferi* induces a robust inflammatory response to recruit leukocytes to the site of infection, repair tissue, and eliminate the infectious factor [13–15]. Infected tissues typically display a mononuclear-type inflammation, with predominating macrophages, dendritic cells, and plasma cells [12, 16]. However, the underlying mechanisms of this inflammatory response and resolution are not clear [13, 14]. The initial innate immune response begins with a signaling cascade which is promoted by cytokines, eicosanoids and related lipid mediators [17, 18]. These lipid mediators are generated from arachidonic acid (AA) after it is enzymatically released from the cell membrane by over 50 different enzymes, which are also capable of metabolizing other polyunsaturated fatty acids (PUFAs) such as linoleic acid (LA) [19, 20]. The two main AA metabolic pathways are the cyclooxygenase pathway (COX-1 and COX-2) which produces prostaglandins (PGs) and thromboxanes (TX), and the lipoxygenase pathway (LOX) which produces leukotrienes (LT), lipoxins (LX), and hydroxyeicosatetraenoic acids (HETE) [14]. These molecules play a role in both the induction as well as the resolution of inflammation, failure of which can lead to prolonged inflammation, severe tissue damage and long-term complications such as arthritis [20].

Although the role of cytokines in the mediation of the inflammatory process in LB has been well described [13], no study has systematically evaluated the role of PGs and related lipid mediators in the induction and resolution of inflammation in LB. Given the high level of inflammation-related long-term effects of LB as well as the widespread use of COX-specific inhibitors and non-steroidal anti-inflammatory drugs (NSAIDs) in the treatment of LB [21], it is critical to understand the role of bioactive lipid mediators in LB to provide better approach for symptoms resolution and LB treatment. The objective of the present review was, therefore, to systematically evaluate the role of AA metabolism and products in LB development.

## Methods

### Literature search

The present systematic review was undertaken and reported in accordance with the PRISMA (Preferred Reporting Items for Systematic Reviews and Meta-Analyses; see Supplementary Table 1) statement [22]. A search was conducted in PubMed, Ovid Medline, and Embase databases using a predefined search strategy (Supplementary Tables 2, 3). Briefly, the following search terms (MeSH) were used: “Lyme” or “*Borrelia burgdorferi*”, and “cyclooxegenase” or “COX” or “prostaglandins” or “eicosanoids” or “arachidonic acids” or “lipoxins” or “leukotriene” or “thromboxanes” or “lipoxygenase” or “prostaglandin synthase” or “eicosapentaenoic acid”. Article publication search was from the inception of the databases to October 23, 2017. Publication dates were not limited in the PubMed search. Only English language articles were included. Review papers, letters to the editor, case-reports, editorials, conference abstracts, vaccine studies, and duplicate studies were excluded. Studies were considered eligible if they investigate the role of PGs or related enzymes and metabolites in LB or reported concentrations of metabolites from the AA pathway and included studies reporting results from cell and animal models as well from humans. Reference lists of included studies were also manually checked for relevant reports for inclusion.

### Inter-reviewer agreement

Two reviewers (AJ, DV) independently reviewed the identified abstracts to identify those eligible for full-paper review and subsequently, inclusion in the present study. Disagreements regarding study inclusion were resolved by an arbitrator (AB). Percentage agreement and Cohen’s Kappa (*К*) statistic and 95% confidence interval (95% CI) were calculated [23]. Interpretation of the agreement between reviewers was based on the following Landis and Koch’s kappa-statistic benchmarks [24]: poor (< 0), slight (0.00–0.20), fair (0.21–0.40), moderate (0.41–0.60), substantial (0.61–0.80), and excellent (0.8–1.0). The agreement on inclusion between the two reviewers was 83%, with moderate κ of 0.54 (95% CI 0.29–0.79).

### Data extraction

Data extracted from the selected studies included the author’s first name, year of publication, and main findings related to products of PGs-related pathways. Data extracted specifically from cell-culture and animal studies included the type of cell or species used, and treatment type, and duration. Data extracted from human studies included the number and group of patient and control subjects, and any reported concentrations of PGs and related products.

## Results

### Search results

**Fig. 1:**
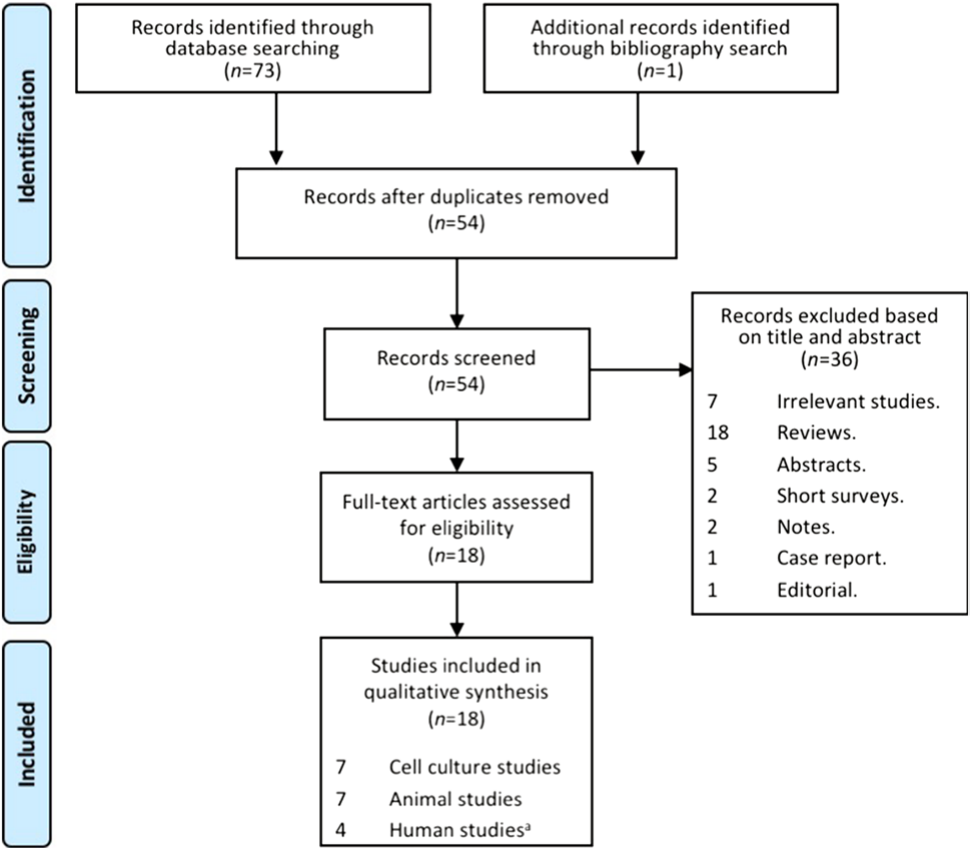
Flowchart of study selection and systematic literature review process. The flow diagram describes the systematic review of literature evaluating the metabolites of prostaglandin synthases as potential biomarkers of Lyme disease severity and symptom resolution. A total of 18 unique studies were identified to be included into the qualitative assessment (7 cell-culture studies, 7 animal studies and 4 studies in human). ^a^Two studies reported the same human population. The study with the larger sample size was included into the qualitative analysis

The systematic literature review process is described in Fig. 1. In the initial database search, 73 studies were identified which met search criteria and were in the English language. Following the removal of 20 duplicate studies, 53 studies remained. One additional study was identified through reference search, for a total of 54 studies that were screened through abstract review. Of these, 36 studies were excluded during abstract review, according to the inclusion and exclusion criteria (Supplementary Table 2). Briefly, 18 review studies, 5 abstracts, 2 short surveys, 2 notes, 1 editorial, and 1 case report were excluded. Additional seven reports were excluded based on their relevance to the present study. Full-text screening was conducted on 18 studies that were selected for final inclusion in the present study. The 18 included studies consisted of 7 cell-culture studies, 7 animal studies, and 4 human studies, which are described in further detail below.

### Cell-culture studies

Seven cell-culture studies were included in the present report, of which four investigated the effects of *B. burgdorferi* infection in brain microglial or astrocytic cells, two in joint cells, and one in splenic cells (Table 1). Microglial-culture studies consistently showed a significant upregulation of COX-2 expression in response to *B. burgdorferi* infection [25–28] with no change in COX-1 expression [26]. These effects may not be similar in astrocytes, where an increase in COX-2 mRNA in response to *B. burgdorferi* infection was observed in one study [27] but not in another [28]. Treatment of *B. burgdorferi*-infected microglia with the NSAID Meloxicam reduced COX-2 activity but was unable to alter COX-2 activity in *B. burgdorferi*-infected astrocytes, suggesting that astrocytes may not respond to *B. burgdorferi* infection by upregulating COX-2 [28]. The increases in COX-2 expression resulted in the elevation in PGE_2_ and EP_2_ expression in murine microglia [25]. This effect was not reproduced in a similar sitting where EP_4_ expression was not altered in response to *B. burgdorferi* infection in microglia [26].

**Table 1:** Summary of cell-culture studies evaluating the role of prostaglandins and related metabolites in Lyme disease

Study	Cell type	Treatment	Hours	Main findings
Rasley et al. [25]	C3H/Hej mice EOC13 microglia and neonatal brain microglia	*B. burgdorferi* exposure (5 µg /ml)	4, 8, 12	- COX2 mRNA was significantly elevated following exposure to *B. burgdorferi* for 8 or 12 h - PGE2 secretion was elevated in both cell types at 12 h post-*B. burgdorferi* exposure
Singh et al. [39]	Synovial cells from normal human joint tissue	*B. burgdorferi* strains: Geho and B31 (MOI 10)	5 (days)	- COX-1 was upregulated with Geho *B. burgdorferi* exposure but not B31 *B. burgdorferi* - PGE_2_ secretion increased with Geho *B. burgdorferi* but decreased with B31 *B. burgdorferi* exposure
Rasley et al. [26]	Mice neonatal brain microglia	*B. burgdorferi* strain N40 (1 µg/ml)	4, 8, 12	- Stimulation with *B. burgdorferi* modestly increased COX-2 expression after 8 h (2.2-fold) but not 4 h (1.3 fold), and EP2 expression at 4 h (18-fold) - There was no change in COX-1, PGE_2_, or EP_4_ expression
Ramesh et al. [27]	*Rhesus macaques *frontal cortex brain tissue	*B. burgdorferi* strain B31 (10 µg/ml)	4, 8	- COX-2 was detected in both astrocytes and microglia in *B. burgdorferi*-infected cells but not in controls
Blaho et al. [29]	Splenic B cells from C3H/HeJ mice (wild-type or COX-1^−/−^)	*B. burgdorferi* (5 µg/ml); with or without COX-1 and COX-2 inhibitors	8, 72	*- B. burgdorferi* increased expression of COX-1 and COX-2 *- B. burgdorferi* increased the levels of PGF_2a_, TXB_2,_ and PGE_2_, which was attenuated by COX-1 or COX-2 inhibitors - FP and TP receptor expression increased with *B. burgdorferi* exposure - COX-1^−/−^ mice produced lower levels of PGF_1α_, TXB_2_, PGF_2α_, PGJ_2_ than WT when exposed to *B. burgdorferi*
Ramesh et al. [28]	Primary *Rhesus macaque *astrocyte and microglia	*B. burgdorferi* strain B31 (10 µg/ml) with and without Meloxicam (100 µM)	24	*- B. burgdorferi* increased COX-2 levels in microglia, while *B. burgdorferi* + Meloxicam reduced COX-2 (*p* < 0.01) *- B. burgdorferi* had no effect on COX-2 levels in astrocytes
Zhang et al. [33]	BMDMs and PMNs from C57BL/6J mouse femurs (WT, 5-LOX^−/−^, BLT1^−/−^, BLT2^−/−^, BLT1/2^−/−^)	*B. burgdorferi* N40 (MOI 10)	1, 4	- 5-LOX^−/−^ and BLT1^−/−^ BMDMs had significantly lower phagocytosis of *B. burgdorferi* than WT, which was restored with LTB_4_ addition - LTB_4_ production was two-to-three times higher in BLT1^−/−^ vs WT BMDMs at 4 h but not 1 h. LTB_4_ production was ten times higher in PMNs vs. BMDMs but did not differ between BLT1^−/−^ vs wild-type animals

Murine splenic cells responded similarly to *B. burgdorferi* infection, with increased expression of COX-1 and COX-2, as well as prostaglandins PGF_2α_ and PGE_2_ (and TBX_2_), but not PGF_1α_ or PGJ_2_ [29]. The rise in PGs levels in response to *B. burgdorferi* appears to be mediated by the COX-pathway, as it was downregulated by either a COX-1 or COX-2 inhibitor, suggesting that both are involved in PGs production during *B. burgdorferi* infection. Furthermore, splenic cells from mice lacking the ability to produce COX-1 produce lower levels of PGF_1α_, PGF_2α_, TXB_2_, and PGJ_2_ than cells from wild-type mice when exposed to *B. burgdorferi* [29]. They also display a defective germinal center (GC) formation, with a reduction in GC size and total splenic area occupied by GC, suggesting that COX-1 may be controlling the production of a key regulator of the GC development process during LB [29].

5-LOX catalyzes the oxygenation of AA into leukotrienes and is expressed predominantly in inflammatory cells such as polymorphonuclear cells (PMNs) [30–32]. Bone marrow-derived macrophages (BMDM’s) from 5-LOX^−/−^ mice had an impaired ability to phagocytose *B. burgdorferi* and take up only about half as many spirochetes as BMDM’s from the wild-type mice [33]. Phagocytic ability of 5-LOX^−/−^ BMDMs was restored with the addition of LTB_4_ [33]. LTB_4_, one of the downstream products of AA-catalysis by 5-LOX, is recognized to attract neutrophils, recruit and activate eosinophils, monocytes, macrophages, mast cells, dendritic cells, and T cells, and is an important mediator of the phagocytosis of several bacterial pathogens [34–36]. LTB_4_ acts through two identified G protein-coupled receptors: the high-affinity BLT1 receptor and the low-affinity BLT2 receptor [37, 38]. Similar to 5-LOX^−/−^ BMDMs, BLT1^−/−^ BMDMs also displayed significantly impaired phagocytosis of *B. burgdorferi* [33]. Phagocytosis was restored by the addition of exogenous LTB_4,_ but not with the concurrent addition of BLT2 antagonist, suggesting the involvement and compensatory capability of the low-affinity BLT2 receptor in LTB_4_-mediated *B. burgdorferi* phagocytosis [33]. Addition of 12(*S*)-hydroxyheptadeca-5Z,8E,12-hydroxyheptadecatrenoic acid (12-HHT), a product of the COX-1 pathway capable of binding BLT2, did not alter phagocytic activity of 5-LOX^−/−^ or BLT1^−/−^ BMDMs, suggesting this activity is specifically regulated by LTB4 [33]. Phagocytosis of *B. burgdorferi* in BLT1^−/−^ PMN’s was only slightly impaired and LTB4 production was ten-fold high than in BLT1^−/−^ BMDM’s [33]. The results of this study suggest the higher LTB4 production in PMN’s may allow for compensatory BLT2-mediated phagocytosis, which does not occur as efficiently in BMDM’s due to their lower production of LTB4.

Interestingly, *B. burgdorferi* effects on COX-1 and PGE_2_ expression in synovial cells may be dependent on the strain of *B. burgdorferi* used. Singh et al. [39] showed human synovial cells upregulated COX-1 in response to exposure to the *B. burgdorferi* sensu stricto isolate Geho but not the isolate B31. Similarly, PGE_2_ secretion increased in response to exposure to Geho isolate, but decreased in response to the B31 *B. burgdorferi* isolate [39]. Therefore, the *B. burgdorferi* strain being evaluated may be an important parameter to consider when comparing the findings of different studies. The *B. burgdorferi* strains used in each report included in the present study are reported in Tables 1 and 2.

**Table 2:** Summary of animal studies evaluating the role of prostaglandins and related metabolites in Lyme disease

Study	Species	Treatment	Days	Main findings
Anguita et al. [40]	C3H mice	*B. burgdorferi* strain N40 (1 × 10^4^ spirochetes); MF-tricyclic COX-2 inhibitor	0–60	- COX-2 expression was elevated following *B. burgdorferi* infection, peaking on day 14 - Mice fed with COX-2 inhibitor showed a reduction in arthritis severity (*p* < 0.01) compared to control mice on day 14 following *B. burgdorferi* infection, but there were no differences in levels of IL-2, IFN-γ, IgG, or IgM
Glasner et al. [41]	C3H/HeJ mice	*B. burgdorferi* strain N40 (final inoculum: 1 × 10^5^)	0–25	- COX-2 expression was significantly elevated, peaking on day 8 post-treatment
Blaho et al. [42]	C3H/HeJ and DBA2/J (arthritis-resistant) mice	*B. burgdorferi* strain N40 (final inoculum: 1 × 10^5^); celecoxib (COX-2 inhibitor)	0–35	- Celecoxib administration resulted in prolonged Lyme arthritis, with a high concentration of infiltrating neutrophils on day 35 post-treatment - Celecoxib administration did not alter IgG or IgM production, or the number of spirochetes in joints. It lowered the upregulation of cPLA_2_, PGE_2_, PGJ_2_ on days 7, 14, and 21, respectively, seen in WT mice
Blaho et al. [29]	C3H, DBA, and COX-2^−/−^ mice	*B. burgdorferi* strain N40 (final inoculum: 1 × 10^5^)	0–35	- Eicosanoid levels were generally elevated in C3H mice, while DBA mice followed similar but muted trends in eicosanoid fluctuation - Some eicosanoids, such as LTB_4_ and PD_1_ were significantly increased during *B. burgdorferi* infection in C3H, but not DBA mice - COX-2^−/−^ mice displayed a dramatic reduction in LTC_4_, LTE_4_, 5-HETE compared to WT following *B. burgdorferi* infection
Blaho et al. [44]	C3H/Hej and 5-LOX^−/−^ mice	*B. burgdorferi* strain N40 (final inoculum: 1 × 10^5^)	0–35	- 5-LOX, FLAP, and LTB_4_ were upregulated in WT ankle joints on days 7–14 post-*B. burgdorferi* infection - 5-LOX^−/−^ mice had greater ankle swelling and arthritis scores than WT on days 7–28 and showed a failure to resolve inflammation compared to WT animals - Spirochete clearance and IgM levels were equal in both groups, but 5-LOX^−/−^ mice had lower IgG levels than WT on day 28 post-treatment
Dumlao et al. [46]	C2H/HeJ mice	*B. burgdorferi* strain N40 (final inoculum: 1 × 10^5^); SO diet- high in n-6 FA’s vs. FO diet high in n-3 FA’s	0–25	- Diet did not have any effect on Lyme arthritis or carditis scores, number of spirochetes, or cellular makeup in day 25 post-treatment - Fish oil (FO) diet resulted in a global shift toward EPA- and DHA-metabolites during *B. burgdorferi* infection vs. SO (soybean oil) diet which resulted in an increase in AA- and LA-derived metabolites
Lasky et al. [45]	C3H and 5-LOX^−/−^ mice	*B. burgdorferi* strain N40 (final inoculum: 1 × 10^5^)	0–56	- M2 and rM macrophages were decreased in the ankle joints of 5-LOX^−/−^ mice on day 21, compared to wild-type. There were no differences in the number of M1 macrophages - In the heart tissue, 5-LOX^−/−^ mice had fewer M2 macrophages, but not M1 or rM macrophages, compared to WT on day 21 post-treatment - By day 56, there were no difference in macrophage subset or number

### Animal studies

Seven animal studies were included in the present systematic review. All studies were conducted on mice where three reports investigated the effect on the COX-2 pathway during *B. burgdorferi* infection and three examined the effect on the 5-LOX pathway. Additionally, one study examined both the effect of *B. burgdorferi* on COX-2 and 5-LOX whereas one study assessed the effect of dietary fatty acid composition on AA-derived metabolites during *B. burgdorferi* infection (see Table 2). COX-2 expression was consistently elevated following *B. burgdorferi* infection in all included studies [40–42]. In a study by Anguita et al. [40], mice fed MF-tricyclic, a COX-2 inhibitor, exhibited a significant reduction in Lyme arthritis severity compared to control mice at 14 days post-*B. burgdorferi* infection. However, this effect did not result in any differences in the levels of IL-2, IFN-γ, IgG, or IgM between inhibitor-administered and non-inhibitor-administered mice [40]. Conversely, Blaho et al. [42] showed that administration of the COX-2 inhibitor celecoxib did not affect arthritis severity scores at the infection peak (day 17) but resulted in prolonged resolution of Lyme arthritis. Although arthritis completely resolved by day 35 in control mice, COX-2 inhibitor-fed mice displayed severe inflammation in their joints at this time, with high arthritis severity scores and a high concentration of infiltrating neutrophils [42].

Celecoxib administration did not alter IgG or IgM production but did reduced the upregulation of cytosolic phospholipases A_2_ (cPLA_2_), PGE_2_, and PGJ_2_ on days 8, 14, and 21, respectively, as seen in wild-type mice [42]. Use of mice lacking the COX-2 gene (COX-2^−/−^) resulted in a similar pattern of an initial inflammatory response similar to wild-type mice and a failure—by day 35—to resolve arthritis [42]. In a follow-up lipidomic analysis of COX-2^−/−^ DBA arthritis-resistant mice, and wild-type mice following *B. burgdorferi* infection, the former animals displayed a significant reduction in PGD_2_ and PGE_2_, as well as 5-LOX products LTC_4_, LTE_4_, and 5-HETE [43]. Arthritis-resistant DBA mice followed similar, but muted, patterns in eicosanoid fluctuation as wild-type mice following *B. burgdorferi* infection. Only some eicosanoids, such as LTB_4_ and PD_1_, which significantly increased during *B. burgdorferi* infection in wild-type mice were not affected in the arthritis-resistant DBA mice [43].

Following *B. burgdorferi* infection, severe inflammation is observed in the ankle joints of wild-type mice on day 14 where 5-LOX, FLAP, and LTB_4_ were significantly upregulated, an effect that was resolved by day 28 [44]. In this study, *B. burgdorferi*-infected 5-LOX^−/−^ mice lacking the 5-LOX gene experienced similarly severe inflammation to wild-type mice on day 14, but this effect was not resolved by day 28 as in wild-type mice [44]. At 60 days post-infection, ankle swelling returned to baseline in wild-type (WT) mice but not in 5-LOX^−/−^ mice, indicating a continued delay in arthritis resolution in the absence of the 5-LOX gene. Joints of 5-LOX^−/−^ mice revealed the continued presence of neutrophils, indicative of an ongoing inflammatory response, while joints from wild-type mice contained mostly macrophages [44]. Sera of 5-LOX^−/−^ mice also contained fewer *B. burgdorferi*-specific IgG antibodies than WT mice by day 28, although spirochete clearance was not affected. Bone marrow-derived neutrophils and macrophages both had impaired ability to phagocytose opsonized and un-opsonized *B. burgdorferi*, respectively [44]. These results suggest the potential involvement of leukotrienes derived from the 5-LOX pathway in macrophage and neutrophil clearance of *B. burgdorferi*, and the resolution of inflammation following *B. burgdorferi* infection. To further investigate the effect of 5-LOX in Lyme arthritis, Lasky et al. [45] identified macrophage subsets in the joints and hearts of 5-LOX^−/−^ mice. Compared to wild-type animals, 5-LOX^−/−^ mice had fewer M2 and rM macrophages in the ankle joints and heart on day 21, i.e., at the peak of inflammation. By the resolution of inflammation on day 56, there were no differences in the subsets or number of macrophages between 5-LOX^−/−^ and wild-type mice [45].

It was noted that dietary composition influences the lipidomic profile of *B. burgdorferi*-infected mice and subsequently the PGs synthesis [46]. Lipidomic analysis of *B. burgdorferi*-infected mice fed a diet high in fish oil (FO), compared to those fed a diet high in soybean oil (SO), revealed a global shift toward EPA- and DHA-derived metabolites [46]. In contrast, mice fed the SO diet experienced a shift toward AA- and LA-derived metabolites. Prostaglandins PGE_3_ and PGD_3_ were higher in FO-diet-fed mice, while 5-HETE was largely stable in FO-fed mice and only elevated in SO-fed *B. burgdorferi*-infected animals [46].

### Human studies

A summary of main findings from human studies and values of the PGs and AA metabolites evaluated in each study is shown in Table 3. Although four human studies were identified and included in the present review, two studies [47, 48] were duplicate from the same patient population. The study population included in the Luczaj et al. [48] was extended by 51 patients and 25 controls to form the study population of the Luczaj et al. study [47]. Therefore, we only considered the latter in our assessment. Of the three remaining human studies, although different PGs were examined, both reported significant differences between LD patients and their comparison groups. Mayatepek et al. [49] directly measured the concentrations of PGs in patients with Lyme meningitis (LM) and Lyme arthritis (LA) vs controls with noninflammatory arthropathy. Compared to controls, patients with LA had significantly higher levels of synovial fluid LTB_4_ but not urinary LTE_4_, PMNL LTB_4_ and LTC_4_ [49]. A variety of AA oxidation products—independent of the COX-pathway (via free-radical induced peroxidation) were also evaluated in LA patients [50]. Higher levels of plasma free AA, 8-isoPGF_2α_ and PLA_2_ activity as well as elevated levels of urinary 8-isoPGF_2α_ were observed in LA patients compared to their health counterparts [47, 48]. LA patients also exhibited lower plasma concentrations of phospholipid AA and free 8-isoPGF_2α_ [47]. Although antibiotic treatment of LA patients resulted in lowering the levels of plasma total 8-isoPGF_2α_, free 8-isoPGF_2α_, PLA_2_ activity, and urinary 8-isoPGF_2α_, these levels remained higher than those of controls [47, 48]. Overall, the lipidomic profile in LB patients showed a shift in six PGs metabolites compared to controls [51].

**Table 3:** Summary of human studies evaluating the role of prostaglandins and related metabolites in Lyme disease

Study	Subjects (*n*)	Mean concentrations of arachidonic acid metabolite or related activity			Findings
		Metabolite/activity	Patients	Controls	
Mayatepek et al. [49]	Lyme meningitis (10) vs. healthy controls (10)	PMNLs LTB_4_ (ng/10^6^ cells)	39.3	35.2	- LTB_4_ concentrations in synovial fluid were significantly higher in patients with Lyme arthritis compared to those with noninflammatory arthropathy (*p* < 0.05). Moreover, concentrations of PMNL LTB_4_ and LTC_4_, and urinary LTE_4_ did not differ between LB patients and controls
		PMNLs LTC_4_ (ng/10^6^ cells)	5.3	6.0	
		Urine LTE_4_ (pmol/l)	273	265	
	Lyme arthritis (7) vs noninflammatory arthropathy controls (7)	PMNLs LTB_4_ (ng/10^6^ cells)	40.0	38.4	
		PMNLs LTC_4_ (ng/10^6^ cells)	4.8	4.6	
		Synovial LTB_4_ (ng/ml)	142	46	
		Urine LTE_4_ (pmol/l)	279	262	
Luczaj et al. [47]	Lyme arthritis (57) vs. healthy controls (41)	Plasma free AA (nmol/µl)	0.203	0.166	- Compared to controls, patients with Lyme arthritis had significantly elevated levels of plasma: free AA, total 8-isoPGF_2α_, and PLA_2_ activity, and urinary 8-isoPGF_2α_. Patients also had significantly lower levels of plasma: phospholipid AA and free 8-isoPGF_2α_ (*p* < 0.05). After antibiotic treatment, patients had significantly reduced levels of plasma: total 8-isoPGF_2α_, free 8-isoPGF_2α_, PLA_2_ activity, and urinary 8-isoPGF_2α_, compared to pre-antibiotic treatment (*p* < 0.05)
		Plasma phospholipid AA (nmol/µl)	24.11	36.86	
		Plasma total 8-isoPGF_2α_ (pg/ml)	52.15	18.99	
		Plasma free 8-isoPGF_2α_ (pg/ml)	3.48	5.16	
		Free 8-isoPGF_2α_ (%)	7	27	
		Urine 8-isoPGF_2α_ (pg/mg creatinine)	206.59	37.04	
		Plasma PLA_2_ activity (nmol/min/ml)	28.68	9.00	
		Plasma PAF-AH activity (nmol/min/ml)	24.17	25.65	
	Lyme arthritis pre-antibiotic treatment (13) vs. Lyme arthritis post-antibiotic treatment (13)	Plasma free AA (nmol/µl)	0.190	0.180	
		Plasma phospholipid AA (nmol/µl)	20.06	21.18	
		Plasma total 8-isoPGF_2α_ (pg/ml)	75.43	48.56	
		Plasma free 8-isoPGF_2α_ (pg/ml)	3.01	2.83	
		Free 8-isoPGF_2α_ (%)	4	6	
		Urine 8-isoPGF_2α_ (pg/mg creatinine)	237.42	144.24	
		Plasma PLA_2_ activity (nmol/min/ml)	31.92	23.51	
		Plasma PAF-AH activity (nmol/min/ml)	23.14	29.85	
Molins et al. [51]	Lyme disease (202) vs healthy controls (158) and other controls (101)	No specific metabolites were reported	–	–	- Compared to controls, lyme disease patients had a shift in the abundance of 49 molecular features, including 6 products of prostaglandin metabolism

## Discussion

The present study was conducted to systematically evaluate the role of PGs and other lipid mediators derived from AA metabolism in LB development and complications. Studies investigating the levels of AA-related products in patients with LB suggest that several PGs and LTB_4_ are significantly elevated compared to controls [49, 51]. At least six PGs were found to distinguish patients with early LB from healthy individuals [51], suggesting that these molecules can be potential targets for the development of novel diagnostic markers for the detection of early LB. Levels of plasma AA and prostaglandin peroxidation product 8-isoPGF_2α_ were also elevated in LA patients compared to controls [48], demonstrating the increased level of oxidative stress that occurs during LA. Antibiotic therapy was effective in reducing the levels of these lipid metabolites, indicating that their production is a dynamic process that can be modulated by therapy. Although 8-isoPGF_2α_ can be implicated as a mediator of LA development, its use as a diagnostic marker may be limited as it is also strongly associated with other types of arthritis [48].

While human studies measuring the levels of AA-derived lipid mediators in LB are limited, results from animal and cell-culture models confirm the observed rises in PGs, thromboxane (TXBs) and leukotriene (LTs) concentration during LB development and progression and provide insight into their pathophysiological implications. COX-2, which is typically undetectable in healthy tissue, has been consistently elevated in murine LA models, as well as microglial and B cells of LB [25–29, 40–42]. Although COX-1 is constitutively expressed in most cells, it is also responsible for the synthesis of PGs and TBXs and was shown to be upregulated in splenic B cells in response to *B. burgdorferi* [26, 29]. The induction in COX-2 expression is a key component of innate immune cell function during the inflammatory phase of the immune response, and both COX-1 and COX-2 play a role in T-cell-mediated immunity [52–54]. The rise in COX-1 and COX-2 expressions and activities in LB was accompanied in most studies by an upregulation of PGs production, including PGE_2_ and PGF_2α_ as well as TXB_2_ and EP_2_ [25, 26, 29, 39]. Blockage of COX-1 and COX-2 function using specific inhibitors was effective in decreasing PGs concentrations in murine B cells [29], and decreased arthritis severity scores in a murine model of LA [40]. COX-1^−/−^ mice similarly experienced reductions in the production of various PGs and TXBs during *B. burgdorferi* infection [29]. These results suggest that the use of COX-inhibiting anti-inflammatory agents may be an effective treatment approach in LB, particularly in LA prevention. However, use of a COX-2 inhibitor was found in one study to delay arthritis resolution, despite being effective in reducing peak levels of cPLA_2_, PGE_2_, PGJ_2_ [42]. Mice lacking the ability to produce COX-2 similarly displayed a failure to resolve LA despite reductions in PGD_2_, PGE_2_, LTC_4_, LTE_4_, and 5-HETE [43], suggesting that these lipid mediators may be crucial for the resolution of inflammation during LB, and its failure may lead to LA and other long-term complications following *B. burgdorferi* infection.

The LOX pathway of AA metabolism has also been implicated in LB in several studies, particularly in the resolution phase of the inflammatory process. 5-LOX, and its product LTB_4,_ were shown to be elevated in the ankle joints of mice during *B. burgdorferi* infection [44]. Similar to the lack of inflammation resolution seen in COX^−/−^ mice, LOX-5^−/−^ failed to resolve ankle swelling, reduced phagocytic capacity, lowered the *B. burgdorferi*-specific IgG, and caused fewer macrophages in the joints and hearts of *B. burgdorferi*-infected mice [44, 45]. LTB_4_ may be a lipid mediator of particular interest, as it has been shown to be elevated in LB patients and the ankle joints of *B. burgdorferi*-infected mice [44, 49], and its addition to 5-LOX^−/−^ BMDMs was able to completely restore their phagocytic capacity [33]. Clearance of apoptotic neutrophils by macrophages is a critical process for the resolution of inflammation during microbial infection and has been shown to involve macrophage production of pro-resolving lipoxins and PGs [55]. M2-macrophages, polarized by IL-4, produce more pro-resolving mediators than M1-macrophages—polarized by IFNγ—which synthesizes more pro-inflammatory mediators [55]. 5-LOX^−/−^ mice have fewer M2-macrophages in their ankle joints and heart tissue than M1-macrophages [45], suggesting that recruitment of M2-macrophages may be a mechanistic pathway of action for the inflammation resolution effect of 5-LOX-derived lipid mediators.

Findings of the present study suggest that metabolites of the COX- and LOX-pathways are elevated in patients with LB. A common therapeutic approach to lowering PGs levels and decreasing inflammation is the use of NSAIDs that inhibit COX-related activities. NSAIDs are commonly used in the clinical treatment of inflammation, including in patients with LB [56]. Indeed, results of animal studies included here suggest that use of NSAIDs may be effective in reducing prostaglandin concentrations and the severity of arthritis severity [29, 40]. NSAIDs are commonly used to treat inflammation in LB and were particularly reported to resolve symptoms of antibiotic-refractory LA in some patients [57]. This use of NSAIDs was found, however, to be accompanied by several adverse effects, most commonly gastric mucosal injury and was deemed not to be safe for all patients, e.g., those at-risk for heart disease [58]. On the other hand, several nutritional factors, such as vitamins D, E, and C, as well as fatty acids DHA and EPA, have been demonstrated to downregulate PGs synthesis in human cells [59–61]. As shown here, dietary composition may influence the lipidomic profile in LB, where a diet high in fish-oil was found to reduce the secretion of PGs and shift the lipidomic profile towards DHA- and EPA-derived metabolites [46]. This may be particularly beneficial to LB patients as DHA- and EPA-derived lipid mediators can prevent infiltration of immune cells into the cite of infection and signal for the phagocytosis of apoptotic immune cells [62, 63]. As these findings have not been replicated in humans, further studies of nutritional modulation of inflammation in LB may present patient and clinicians with a low-risk complementary treatment approach to current therapies.

Although the present study provides a novel and comprehensive evaluation of the available literature on the role of AA-derived lipid mediators in LB, several limitations exist. Only four human studies from three sets of subjects were identified which—along with the lack of duplicating these findings—weakens the reliability and consistency of their conclusions and outcomes. Furthermore, this small number of human studies substantiating a role of AA-metabolites in LB have a small number of subjects, limited range of lipid mediators and non-inclusive LB stages. Although animal and cell-culture models suggest the involvement of several PGs, TXBs, and LTs in LB, many of these molecules have not been assessed in humans which questions the efficacy of their modulation in symptoms resolution in LB patients. Several COX and LOX-derived lipid mediators are shown to be involved in the resolution phase of the inflammatory process in the LB-animal models. Although these metabolites are known to be generally involved in inflammation resolution in humans [64], no human studies exist to confirm their role in the resolution of inflammation during LD progression.

In conclusion, similar to the cytokines/chemokines-related pathways [13], dysregulation of PGs synthesis, and its associated lipid mediators, seems to play a role in the etiology of LB and the subsequent persistence of inflammation. Failure to resolve this inflammation may lead to long-term complications, including LA. Though these complications usually resolve following appropriate antibiotic therapy, some patients experience persistent symptoms termed ‘antibiotic-refractory LA’ that are accompanied by a slow resolution of inflammation in the joints [12]. The causes of these persistent symptoms are yet to be fully characterized, and consequently, adequate treatment is not available [12]. The present review indicates that COX- and LOX-derived lipid mediators may be a crucial part of the resolution of inflammation in LB and the subsequent LA. Although studies are needed to confirm these findings in LB, future investigations are warranted to address the pathophysiological mechanisms driving antibiotic-refractory LA and identify therapeutic targets. Furthermore, studies assessing the role of AA-derived lipid mediators in humans suggest that the concentrations of several prostaglandins may be used to differentiate early LB patients from healthy individuals. Further investigation into the precise levels of a wide range of PGs, TXBs, and LTs is critical to provide more insight into the nature of the inflammatory process during early LB and may yield novel markers that can be used for early diagnosis. Existing LB diagnostic methods have a limited sensitivity for identifying early LB patients [9]. As such, novel diagnostic tools enabling early LB diagnosis and intervention would have a great impact on patient outcomes.

## Electronic supplementary material

Below is the link to the electronic supplementary material.

Supplementary material 1 (PDF 264 KB)
