# Supplementary material for: Metabolites of prostaglandin synthases as potential biomarkers of Lyme disease severity and symptom resolution
Source: Inflamm Res. 2018 Aug 18;68(1):7–17. doi: 10.1007/s00011-018-1180-5 (PMC6314976; doi:10.1007/s00011-018-1180-5)
Supplement: Supplementary file 1 — Supplementary material 1 (PDF 264 KB) [file 11_2018_1180_MOESM1_ESM.pdf]

Supplementary Table 1. PRISMA Checklist [22].

Section/topic	#	Checklist item	page #
		**TITLE**	
Title	1	Identify the report as a systematic review, meta-analysis, or both.	1
		**ABSTRACT**	
Structured summary	2	Provide a structured summary including, as applicable: background; objectives; data sources; study eligibility criteria, participants, and interventions; study appraisal and synthesis methods; results; limitations; conclusions and implications of key findings; systematic review registration number.	2
		**INTRODUCTION**	
Rationale	3	Describe the rationale for the review in the context of what is already known.	2-4
Objectives	4	Provide an explicit statement of questions being addressed with reference to participants, interventions, comparisons, outcomes, and study design (PICOS).	Sup Table 2
		**METHODS**	
Protocol and registration	5	Indicate if a review protocol exists, if and where it can be accessed (e.g., Web address), and, if available, provide registration information including registration number.	No
Eligibility criteria	6	Specify study characteristics (e.g., PICOS, length of follow-up) and report characteristics (e.g., years considered, language, publication status) used as criteria for eligibility, giving rationale.	5
Information sources	7	Describe all information sources (e.g., databases with dates of coverage, contact with study authors to identify additional studies) in the search and date last searched.	5
Search	8	Present full electronic search strategy for at least one database, including any limits used, such that it could be repeated.	Sup Table 3
Study selection	9	State the process for selecting studies (i.e., screening, eligibility, included in systematic review, and, if applicable, included in the meta-analysis).	Sup Table 3
Data collection process	10	Describe method of data extraction from reports (e.g. piloted forms, independently, in duplicate) and any processes for obtaining and confirming data from investigators.	6
Data items	11	List and define all variables for which data were sought (e.g. PICOS, funding sources) and any assumptions and simplifications made.	6
Risk of bias in individual studies	12	Describe methods used for assessing risk of bias of individual studies (including specification of whether this was done at the study or outcome level), and how this information is to be used in data synthesis.	6
Summary measures	13	State the principal summary measures (e.g. risk ratio, difference in means).	6
Synthesis of results	14	Describe the methods of handling data and combining results of studies, if done, including measures of consistency (e.g. I^2) for each meta-analysis.	None
Risk of bias across studies	15	Specify any assessment of risk of bias that may affect the cumulative evidence (e.g. publication bias, selective reporting within studies).	None
Additional analyses	16	Describe methods of additional analyses (e.g. sensitivity or subgroup analysis, meta-regression), if done, indicating which were pre-specified	None
		**RESULTS**	
Study selection	17	Give numbers of studies screened, assess for eligibility, and included in the review, with reasons for exclusion at each stage, ideally with a flow diagram.	Figure 1
Study characteristics	18	For each study, present characteristics for which data were extracted (e.g. study size, PICOS, follow-up period) and provide the citations.	Tables 1-3
Risk of bias within studies	19	Present data on risk of bias of each study and, if available, any outcome level assessment (see item 12).	None
Results of individual studies	20	For all outcomes considered (benefits or harms), present, for each study: (a) simple summary data for each intervention group (b) effect estimates and confidence intervals, ideally with forest plot.	None
Synthesis of results	21	Present results of each meta-analysis done, including confidence intervals and measures of consistency.	None
Risk of bias across studies	22	Present results of any assessment of risk of bias across studies (see Item 15)	None
Additional analysis	23	Give results of additional analyses, if done (e.g. sensitivity or subgroup analyses, meta-regression [see item 16]).	None
		**DISCUSSION**	
Summary of evidence	24	Summarize the main findings including the strength of evidence for each main outcome; consider their relevance to key groups (e.g., healthcare providers, users, and policy makers).	14-18
Limitations	25	Discuss limitations at study and outcome level (e.g., risk of bias), and at review-level (e.g., incomplete retrieval of identified research, reporting bias).	18
Conclusions	26	Provide a general interpretation of the results in the context of other evidence, and implications for future research.	18
		**FUNDING**	
Funding	27	Describe sources of funding for the systematic review and other support (e.g., supply of data); role of funders for the systematic review.	19

Supplementary Table 2. PICOST Table

Component	Criteria
**Population** (Lyme disease)	Individuals with Lyme disease, animal models of Lyme disease or cell cultures treated for Lyme disease, at any stage
**Intervention**	No intervention criteria
**Comparison**	– No comparison<br>– May compare Lyme disease patients to healthy controls or those with other infectious diseases<br>– Animal models may compare normal strains with knockout Lyme-resistant strains
**Outcome**	– Concentration of prostaglandins and other products of arachidonic acid metabolism in any stage of Lyme disease<br>– Changes in Lyme disease progression or resolution when the prostaglandin H-synthase pathway is impaired or upregulated
**Study design**	English-language studies:<br>– Observational studies (including retrospective chart review)<br>– Case-control studies<br>– Case-studies<br>– Cohort studies<br>– Randomized Control Trial<br>– Cross-sectional studies<br>– In vitro studies<br>– Ex vivo studies<br>– In vivo studies<br>– Experimental studies
**Time**	No time restriction
**Selection criteria for full text screening**	**Inclusions:**<br>– Human studies: patients currently diagnosed with Lyme disease, at any stage. Comparison group not necessary<br>– Animal Studies: any animal model of Lyme disease which evaluates the role of prostaglandins or related enzymes and metabolites<br>– Cell-culture studies: may use human or animal cells to investigate the role of prostaglandins or related enzymes and metabolites in Lyme disease<br><br>**Exclusions:**<br>– Review articles, letters to the editor, case-reports, editorials, conference abstracts<br>– Duplicate study<br>– Non-English

Supplementary Table 3. Specific search strategies.

Category	#	Searches	Results
Details: Ovid MEDLINE(R) <1946 to October Week 2 2017>, Ovid MEDLINE(R) Epub Ahead of Print <October 23, 2017>, Embase Classic+Embase <1947 to 2017 October 23>			
Search terms	1	exp lyme/	28883
	2	exp Borrelia burgdorferi/	20985
	3	1 or 2	33597
	4	exp cyclooxygenase /	130318
	5	exp COX /	320709
	6	exp prostaglandins /	101349
	7	exp eicosanoids/	16027
	8	exp Arachidonic acids/	19094
	9	exp Lipoxin/	2261
	10	exp leukotrienes /	18685
	11	exp thromboxanes /	4918
	12	exp lipoxygenase /	40423
	13	exp prostaglandin synthase/	26284
	14	exp Eicosapentaenoic Acid/	19206
	15	4 or 5 or 6 or 7 or 8 or 9 or 10 or 11 or 12 or 13 or 14	558318
Combination	16	3 and 15	80
Limitation	17	limit 16 to English language	73
De-duplication	18	remove duplicates from 17	53
